# Genetic diversity and wing geometric morphometrics among four populations of *Aedes aegypti* (Diptera: Culicidae) from Benin

**DOI:** 10.1186/s13071-023-05943-6

**Published:** 2023-09-09

**Authors:** Gildas Hounkanrin, Carine Tchibozo, Felix Gregor Sauer, Eric Agboli, Jonas Schmidt-Chanasit, Anges Yadouleton, Renke Lühken, Hanna Jöst

**Affiliations:** 1 Laboratory of Viral Haemorrhagic Fevers and Arboviruses of Benin, Cotonou, Benin; 2 Bernhard Nocht Institute for Tropical Medicine, WHO Collaborating Centre for Arbovirus and Haemorrhagic Fever Reference and Research, Hamburg, Germany; 3 School of Basic and Biomedical Sciences, University of Health and Allied Sciences, Ho, Ghana; 4 Faculty of Mathematics, Informatics and Natural Sciences, Universität Hamburg, Hamburg, Germany; 5 Centre de Recherche Entomologique de Cotonou, Cotonou, Benin; 6 Ecole Normale Supérieure de Natitingou, National University of Science, Technology, Engineering and Mathematics, Abomey, Benin

**Keywords:** *Aedes aegypti*, Africa, Benin, Genetics, Morphometry, Population structure

## Abstract

**Background:**

The impact of the arbovirus vector *Aedes aegypti* is of major concern for global public health as the viruses that it transmits affect millions of people each year worldwide. Originating in Africa, *Ae. aegypti* has now spread throughout much of the world. While the genetic makeup of *Ae. aegypti* in the New World has been extensively studied, there is limited knowledge on its genetic diversity in Africa, particularly at a microgeographical level.

**Methods:**

We investigated mitochondrial cytochrome oxidase I of four *Ae. aegypti* populations from Benin and employed wing morphometric analyses as a cost-effective and reliable tool to explore population structure. Our sampling encompassed various areas of Benin, from the southern to the northern borders of the country, and included urban, semi-urban, and sylvatic sites.

**Results:**

We observed a notable level of genetic diversity (haplotype diversity of 0.8333) and nucleotide diversity (0.00421986), and identified seven distinct haplotypes. Sylvatic and semi-urban sites exhibited a greater number of haplotypes compared to urban sites. Utilizing 18 wing landmarks, we calculated the centroid size, which revealed significant variation among the three landscape types. However, principal component analysis, employed to assess wing shape variation, did not demonstrate significant differences between populations based on landscape type.

**Conclusions:**

Our findings indicate substantial genetic and morphological diversity among *Ae. aegypti* populations in Benin, and provide insight into important biological characteristics of these populations with respect to their potential to transmit viruses. To the best of our knowledge, this is the first study undertaken in Africa to integrate genetics with morphology to analyse the population structure of the major arbovirus vector *Ae. aegypti*.

**Graphical Abstract:**

**Figure:**
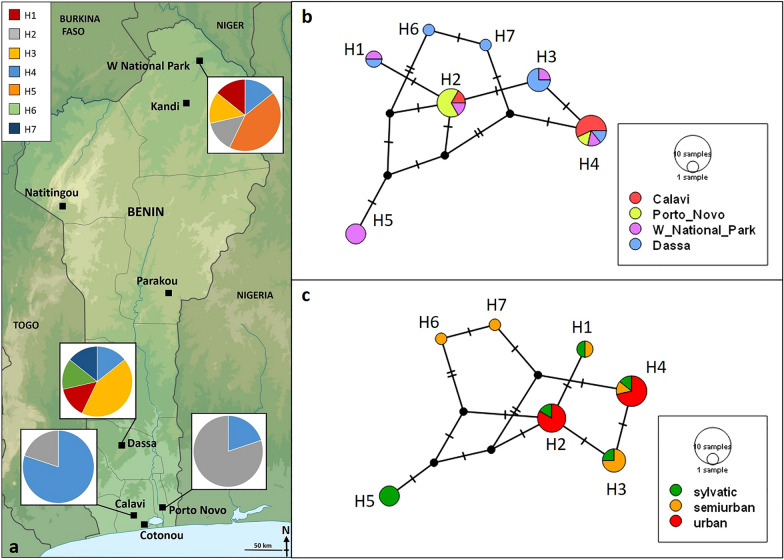

**Supplementary Information:**

The online version contains supplementary material available at 10.1186/s13071-023-05943-6.

## Background

*Aedes aegypti* (Linnaeus, 1762) is a significant global vector responsible for transmitting arboviruses. Originally hailing from Africa, this species is found in tropical and subtropical regions, particularly in urban areas, and serves as a primary vector for dengue, yellow fever, chikungunya, and Zika viruses [1]. Historically, *Ae. aegypti* (subspecies *Aedes aegypti* *formosus*) preferred non-human hosts and inhabited tropical forests, with larvae breeding in tree holes. However, as human populations expanded in Africa, *Ae. aegypti* populations underwent evolutionary changes to adapt to human habitats. These changes involved the use of artificial water containers as larval habitats and a preference for humans as blood meal sources [2]. The human-associated form (subspecies *Aedes aegypti* *aegypti*) was introduced into the New World approximately 500 years ago through slave trade ships originating from Africa [3]. Initially, the subspecies were distinguished based on body colour and scaling patterns on the first abdominal tergite, with *Ae. aegypti formosus* exhibiting darker body colour and *Ae. aegypti aegypti* displaying lighter body colour [4]. However, scale patterns have proven to be highly genetically variable within and between populations of *Ae. aegypti* occupying different ecological niches, which makes the use of these patterns for subspecies discrimination challenging [5].

Genetic data clearly demonstrate a significant genetic differentiation between ancestral African populations of *Ae. aegypti* and populations found outside of Africa. Extensive studies have examined populations outside of Africa, revealing distinct genetic structures within and across continents, indicating high genetic diversity even at a microgeographical scale [6]. However, our understanding of the population structure of *Ae. aegypti*, particularly in West Africa, remains limited [7]. Kotsakiozi et al. [8] and Gloria-Soria et al. [9] explored populations from various African countries and identified two major genetic groups corresponding to west–east differentiation. These studies also revealed long-distance migration with limited local migration, leading to significant isolation by distance. Nonetheless, population structures of *Ae. aegypti* at finer scales remain poorly understood. Understanding the factors that influence the population structure of *Ae. aegypti* can aid in the prevention and control of the diseases that it transmits, and preparedness for potential future threats posed by this species.

The genetics of mosquito species are known to influence important traits such as vector competence, which in turn affect the potential for transmission, spread and establishment of arboviruses. Intraspecific genetic diversity is primarily shaped by fluctuations in population size [10], which, in turn, are influenced by factors such as habitat stability, urbanization, water storage, vector control, and environmental conditions like land use, temperature, relative humidity, and precipitation [11, 12]. Population genetics data can provide insights into population responses to selective pressures. Mitochondrial DNA, particularly fragments of cytochrome oxidase I (COI), has become a widely used molecular marker for species gene flow, and COI is frequently employed in population genetic studies, including those focused on *Ae. aegypti* [13, 14]. The advantages of its use lie in its simple structure, rapid evolution, and abundance in cells, which simplifies COI studies and enables comparison within and between species.

Morphological analysis, specifically that of geometric characteristics, offers another monitoring tool for the study of inter- and intraspecific variation. Wing size and shape are known to be the first morphological traits influenced by environmental and genetic factors [15, 16]. Morales-Vargas et al. [17] investigated populations of *Aedes albopictus* in Thailand and found that wing size is influenced by climatic factors, while wing shape provides insights into heritable intraspecific and geographic differences. Geometric morphometric (GM) analysis provides information on phenotypic biomarkers which, when combined with genetic data, can provide precise information on population structure. Furthermore, GM analysis can aid in the prediction of critical biological characteristics of mosquitoes, such as flight capacity, gamete production, and virus transmission potential [18]. GM studies conducted on *Ae. aegypti* populations in Brazil and Thailand revealed associations between population structure and degrees of urbanization and land use [16, 19].

In Benin, West Africa, two distinct biogeographic zones can be identified: the Sudanian zone in the north, characterized by an annual rainfall of 600–1200 mm, and the Guinean zone in the south and centre, with an average annual rainfall of 1200–2200 mm. *Aedes aegypti* is recognized as the primary vector of dengue virus in Benin, but there is limited information on the burden of this disease there due to transmission by this vector [20, 21]. The aim of our study was to analyse genetic and GM data of *Ae. aegypti* to investigate the influence of geography and landscape type on its populations across multiple regions in Benin. The results should contribute to a better understanding and characterization of local adaptations in this crucial vector species, which could ultimately inform targeted vector control measures.

## Methods

**Fig. 1:**
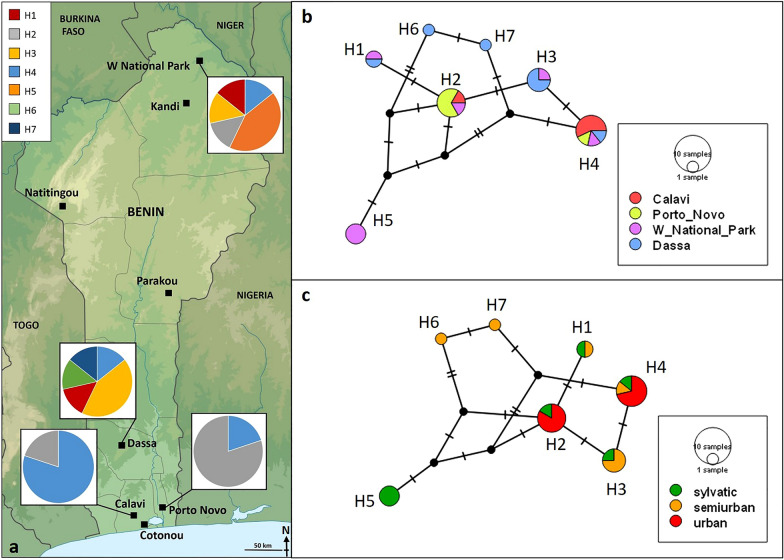
**a** Map of Benin showing the proportions of seven mitochondrial haplotypes (H1–H7) of *Aedes aegypti* by sampling site. **b** Haplotype network based on 24 mitochondrial DNA cytochrome oxidase I sequences of *Ae. aegypti* collected at four sites in Benin by sampling site. **c** Haplotype network by landscape type. Perpendicular bars indicate the number of nucleotide polymorphisms between haplotypes. The circle size indicates the number of individuals sharing the same haplotype

From May 2021 to March 2022, adult *Ae. aegypti* mosquitoes were collected from four sites in Benin (Fig. 1). Three of the trapping sites are located in the Guinean zone: Calavi (6.418736°N, 2.3425287°E; urban), Dassa (7.783625°N, 2.185264°E; semi-urban), and Porto Novo (6.510439°N, 2.604147°E; urban). The fourth trapping site, W National Park (12.040653°, 3.034178°), is situated in the Sudanian zone, and is sylvatic in nature.

The Guinean zone experiences a tropical climate with alternating rainy seasons (April–July and September–October) and dry seasons. Human activities, especially slash-and-burn agriculture, have modified woodland and wooded savannas in this region. Calavi, located in the Atlantique Department, is approximately 18 km north of Cotonou. The trapping site was within the botanical garden of the University Abomey-Calavi, which is surrounded by an urban environment and a lush flora and rich fauna. Porto Novo, an urban trapping site, is characterized by densely populated neighbourhoods with sparse vegetation and limited animal presence. Dassa, the capital of the Collines Department, is situated on a peneplain covered by areas of savanna, trees, shrubs, and intermittent deciduous or semi-deciduous forests. The trapping was conducted on a property on the outskirts of the city, which had abundant vegetation and trees. The northernmost trapping location, W National Park, is a wooded savanna in the Sudanian region. This region, located south of the Sahel, is characterized by isolated trees and wooded savannas, with a dry season lasting 5–7 months (typically May–October). Mosquitoes were captured at a sylvatic site within the wooded savannas, approximately 3 km from the nearest town. Adult mosquitoes were collected with BG-Sentinel mosquito traps (Biogents, Regensburg, Germany). The traps were equipped with BG-Lure (Biogents, Regensburg, Germany) as an attractant and were set in the afternoon for 24 h.

Specimens were morphologically identified using the key of Becker et al. [22]. For further analysis, well-preserved females were selected and checked for white scales on the first abdominal tergite. For GM, specimens were selected randomly from Calavi (16), Dassa (35), Porto Novo (20) and W National Park (12). The right wing was removed and mounted under a cover slip (15 × 15 mm) with Euparal (Carl Roth, Karlsruhe, Germany). Pictures of the right wing were taken under 20× magnification with a stereomicroscope (Bresser Researcher ICD LED 20×–80×; Bresser, Rhede, Germany) and 18 landmarks were digitized with Fiji [23]. The sampling data, landmark coordinates, position and order of landmarks on the  *Aedes aegypti *wing could be found in the Additional file 1: Table S1 and Additional file 2: Fig. S1. Landmarks were determined by one person (GH). A generalized Procrustes analysis of the raw two-dimensional landmark coordinates was performed to calculate the centroid size (CS) and to create the aligned shape coordinates per specimen by using the gpagen function in the R package geomorph [24]. The CS is determined by the square root of the total squared distances measured from the centroid to each of the landmarks, and can be used as a proxy for wing size [25]. ANOVA was used to statistically compare the mean CS of the specimens with landscape type (urban, semi-urban, sylvatic) as the dependent variable. In addition, a Procrustes ANOVA was conducted with the procD.lm function using 1000 permutations to statistically compare the wing shape variation between the three different landscape types [24]. The wing shape variance among the specimens was visualised with a principal component analysis.

For the genetic analysis, 10 specimens from each trapping site were selected and DNA was extracted from one leg. Individual legs were titrated with 500 µl of cell culture medium (high-glucose Dulbecco’s modified Eagle’s medium; Sigma-Aldrich, St. Louis, MO) and zirconia beads (2 mm; Carl Roth). Legs were homogenized for 4 min in a vortexer. The suspension was clarified by centrifugation for 1 min at 8000 r.p.m. and 4 °C, and DNA was extracted with a QIAamp viral RNA mini kit according to the manufacturer’s protocol (Qiagen, Hilden, Germany). A fragment of the COI region with about 710 base pairs was amplified using the primers LCO1490 (5ʹ-GGTCAACAAATCATAAAGATATTGG-3ʹ) and HCO2198 (5ʹ-TAAACTTCAGGGTGACCAAAAAATCA-3ʹ) (5). Polymerase chain reaction products were subjected to Sanger sequencing. Sequences were processed and aligned with Geneious 9.1.8 (Biomatters, Auckland, New Zealand) and trimmed to 501 base pairs. Sequences were submitted to the National Center for Biotechnology Information GenBank and the Basic Local Alignment Search Tool was used to compare the nucleotide data with previously reported sequences. The number of haplotypes, haplotype diversity, nucleotide diversity and Tajima's *D* were computed with DnaSP 10.0.19045 [27]. To construct the haplotype networks, the integer neighbour-joining method was used in Popart software [28].

## Results and discussion

We examined the genetic and morphometric characteristics of four *Ae. aegypti* populations in Benin. The specimens were carefully examined for white scales on the first abdominal tergite, but none were found. Therefore, based on their morphology alone, the analysed specimens were classified as *Ae. aegypti formosus*. A total of 24 COI sequences were obtained and uploaded to GenBank (accession numbers OQ991342-OQ991365). Genetic variability analysis of the 24 COI gene sequences revealed that 494 out of 501 (98.6%) sites were identical. Seven segregating sites defined seven distinct haplotypes (H1–H7), indicating a high level of genetic diversity (haplotype diversity of 0.8333) and nucleotide diversity (0.00421986). The most prevalent haplotype, H4 (29.16%), was found across all four sampling sites (Fig. 1a). Haplotype H2 (25.0%) was detected at Calavi, Porto Novo, and W National Park, while H3 (16.67%) and H1 (8.33%) were found at W National Park and Dassa. H5 (12.5%) was only present at W National Park. Only one specimen each of H6 (4.17%) and H7 (4.17%) were found at Dassa. The number of haplotypes observed at the urban sites Calavi and Porto Novo (*n* = 2) was lower than the number observed at the semi-urban site Dassa (*n* = 5) and the sylvatic site W National Park (*n* = 5). Tajima's* D* statistics were generally positive but not statistically significant (*D* = − 0.420662, *P* > 0.05). Positive values of these statistics may indicate balancing selection or a recent population contraction. A summary of the statistics for COI gene polymorphism by location is given in Additional file 3: Table S2. H5, found at the sylvatic site in the north, exhibited the greatest genetic distance, with a maximum of five segregating sites (Fig. 1b). The urban haplotypes H2 and H4 were more closely related compared to the semi-urban and sylvatic haplotypes (Fig. 1c).

Compared to other species in the family Culicidae, *Ae. aegypti* displays high genetic diversity. Gloria-Soria et al. [9] showed that there is enormous genetic variability and remarkable differentiation between its sylvatic (*Ae. aegypti formosus*) and domestic (*Ae. aegypti aegypti*) subspecies in Africa. It exhibits rapid evolution and adaptability to various ecological conditions [6]. In the present study, we observed high genetic diversity at a microgeographical scale, with greater diversity observed at the semi-urban and sylvatic sites in northern Benin than at the urban sites in the south. Several hypotheses can be proposed to explain these findings, although they should be interpreted with caution due to the limited size of the dataset. (i) The higher genetic diversity at semi-urban and sylvatic sites may be attributable to greater habitat diversity. The habitat heterogeneity hypothesis proposes that variation in environmental factors leads to the provision of more niches, which supports increased intraspecific genetic diversity [29]. (ii) Limited gene flow between populations in the north may occur due to the dry climate, with the dry season lasting approximately 5–7 months, which restricts mosquito presence to sites where humans store water [5]. (iii) Urbanization may lead to increased connectivity among urban sites, resulting in genetic drift or gene flow within a population [30]. (iv) The use of insecticides in cities may exert selection pressure on resistant haplotypes, leading to a reduction in genetic variability within populations [31]. To validate these hypotheses, further studies, particularly those with a focus on the fine-scale distribution of populations in Africa, are especially warranted.

**Fig. 2:**
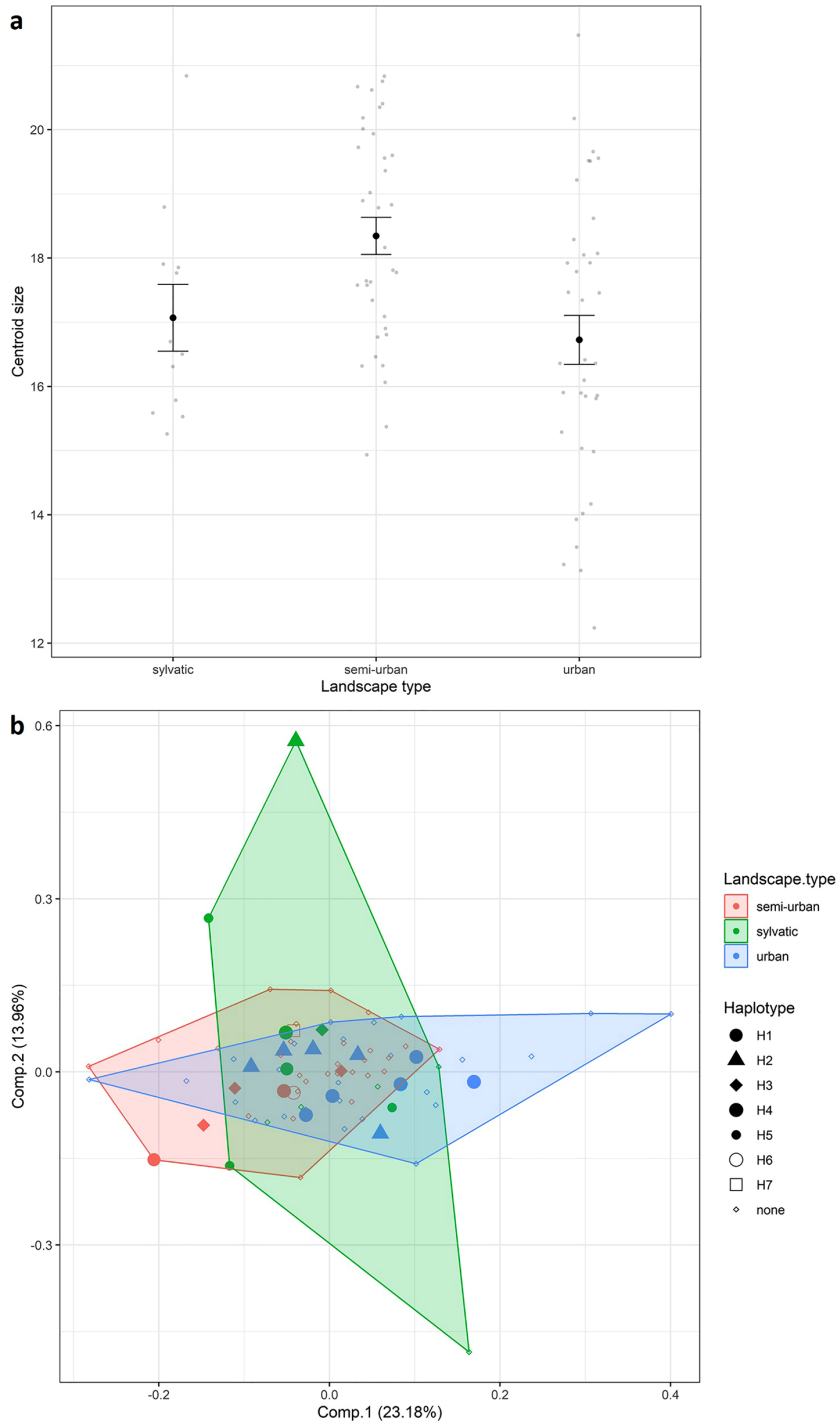
**a** Variation of the centroid size of *Aedes aegypti* specimens per landscape type. **b** Principal component analysis of wing shape variation with haplotype

The wing CS estimated from the coordinates of 18 wing landmarks was utilized to assess wing size and compare the variation in this among different landscape types (Fig. 2; Additional file 1: Table S1). The mean CS exhibited a significant difference among specimens from the various landscape types (ANOVA *F*_2.80_ = 6.419, *P* = 0.0026). The semi-urban site displayed the highest CS, while the urban sites had the lowest. Regarding wing shape, there was only a tendency for a difference among landscape types, which was not statistically significant (ANOVA *F*_2.80_ = 1.5056, *P* = 0.08) (Fig. 2). In certain species, wing shape has been employed as an indicator of population structure, whereas wing size tends to be more sensitive to environmental changes [18]. Morales-Vargas et al. [32] showed that mosquito wing size is influenced by climatic factors, as under natural conditions it is not solely dependent on larval development but also linked to relative humidity during the period of embryonic development. These findings may help to explain our results, i.e. that the variation in wing size in the present study is a consequence of larval habitat quality and differences in relative humidity observed among the three landscape types. We were unable to identify an association between wing shape and haplotypes in our limited dataset (Fig. 2), and we did not analyse the genes responsible for wing shape. Studies investigating the genetic basis of wing shape are scarce and have primarily focused on the model organism* Drosophila melanogaster*. Some genes associated with wing shape have been found to act through growth factor signalling pathways [33].

Another approach used to assess the morphological diversity of populations is to measure the ‘amount of dispersion’ of individuals within a population using principal component analysis, as proposed by Petersen et al. [34]. The polygon formed by the dispersion can serve as an estimator of population diversity and be compared to genetic diversity indices such as haplotype or nucleotide diversity. In our study, the polygon formed using specimens from the sylvatic site was larger than those formed for the other sites. This greater morphological diversity aligns with the higher number of haplotypes found at the sylvatic site. It is important, however, to interpret these results with caution, as geometric morphometrics often fail to demonstrate clear patterns of population structure, and our study was limited to the use of a small sample size. Numerous other factors can influence wing shape and the genetic structure of populations, making the interpretation of results challenging. Nonetheless, the findings of our study provide evidence that *Ae. aegypti* exhibits high genetic and morphological diversity at the microgeographical level, and thus contribute to a better understanding of the biology of this medically significant vector.

### Supplementary Information

**Additional file 1. Table S1**: Sampling data and landmark coordinates.

**Additional file 2. Figure S1**: Position and order of landmarks on *Aedes aegypti* wing.

**Additional file 3. Table S2**: Summary statistics for cytochrome oxidase I (COI) gene polymorphism by location.

## Data Availability

The data generated or analysed during this study are included in this published article and its supplementary information files. Pictures of wings used for the GM analysis have been uploaded to the open data publishing platform Dryad.

## Abbreviations

CS: Centroid size
COI: Cytochrome oxidase I
GM: Geometric morphometric
h: Haplotypes
