# Supplementary figures and images for: Genetic diversity and wing geometric morphometrics among four populations of Aedes aegypti (Diptera: Culicidae) from Benin
Source: Parasit Vectors. 2023 Sep 9;16:320. doi: 10.1186/s13071-023-05943-6 (PMC10492319; doi:10.1186/s13071-023-05943-6)

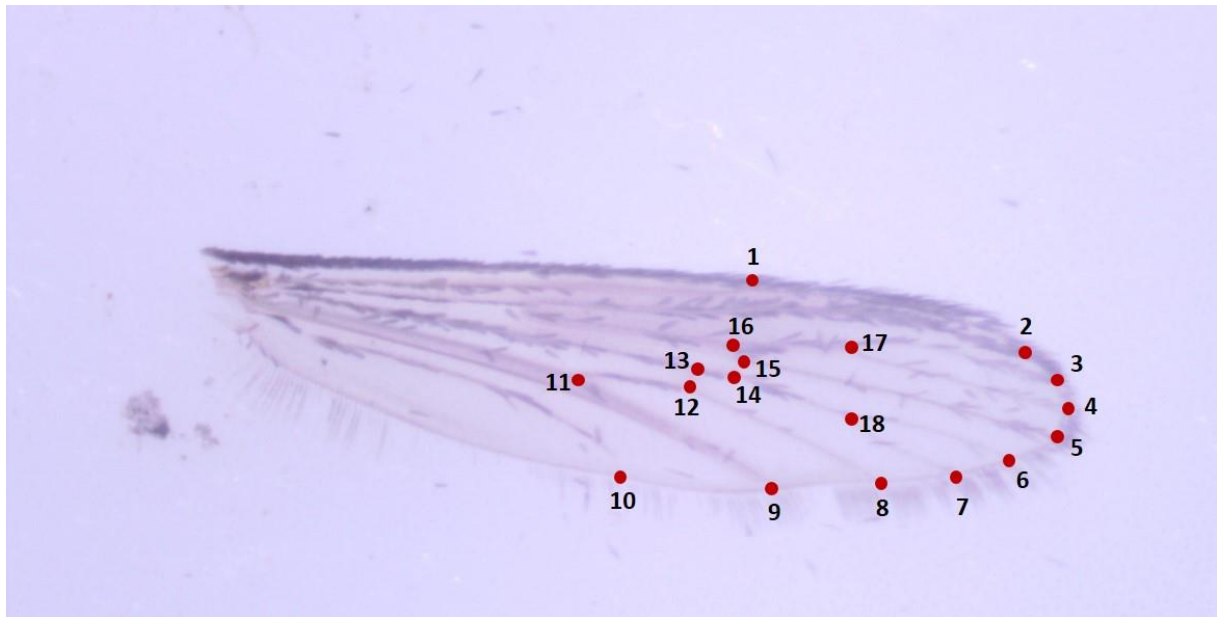

Additional file 2: Figure S1. Position and order of landmarks on *Aedes aegypti* wing.

Supplement: Supplementary file 2 — Additional file 2. Figure S1: Position and order of landmarks on Aedes aegypti wing. [file 13071_2023_5943_MOESM2_ESM.pdf]
