# Supplementary material for: Genetic diversity and wing geometric morphometrics among four populations of Aedes aegypti (Diptera: Culicidae) from Benin
Source: Parasit Vectors. 2023 Sep 9;16:320. doi: 10.1186/s13071-023-05943-6 (PMC10492319; doi:10.1186/s13071-023-05943-6)
Supplement: Supplementary file 3 — Additional file 3. Table S2: Summary statistics for cytochrome oxidase I (COI) gene polymorphism by location. [file 13071_2023_5943_MOESM3_ESM.pdf]

Table S2. Summary statistics for COI gene polymorphism by location.

Location	N	H	S	Hd	Π (k)	D	D*	Fs	F*
Calavi	5	H2, H4	2	0,400	0,00160 (0,800)	-0,97256 ^{ns}	-0,97256^{ns}	1,040 ^{ns}	- 0,95440^{ns}
Dassa	7	H1, H3, H4, H6, H7	5	0,857	0,00456 (2,286)	0,59446^{ns}	0,82622^{ns}	-1,262^{ns}	0,78084^{ns}
Porto Novo	5	H2, H4	2	0,400	0,00160 (0,800)	-0,97256^{ns}	-0,97256^{ns}	- 1,040^{ns}	0,97256^{ns}
W national Park	7	H1, H2, H3, H4, H5	6	0.857	0,00555 (2,762)	0,64916^{ns}	0,43876^{ns}	-0,871^{ns}	0,48985^{ns}
**Total**	**24**	**H1, H2, H3, H4, H5, H6, H7**	**7**	**0,833**	**0,00425 (2,130)**	**0,42670^{ns}**	**1,28730^{ns}**	**-0,649^{ns}**	**1,10964^{ns}**

N, number of sequences; H, haplotype; S, number of polymorphic sites; Hd, haplotype diversity; π, nucleotide diversity; k, mean number of nucleotide differences; D, Tajima statistic; D* and F*, Fu and Li statistics; Fs, Fu statistic; ns, not significant
